# Theoretical Study of the Effect of Different π Bridges Including an Azomethine Group in Triphenylamine-Based Dye for Dye-Sensitized Solar Cells

**DOI:** 10.3390/molecules24213897

**Published:** 2019-10-29

**Authors:** Tomás Delgado-Montiel, Rody Soto-Rojo, Jesús Baldenebro-López, Daniel Glossman-Mitnik

**Affiliations:** 1 Facultad de Ingeniería Mochis, Universidad Autónoma de Sinaloa, Prol. Ángel Flores y Fuente de Poseidón, S/N, Los Mochis 81223, Sinaloa, Mexico; tomas.delgado@uas.edu.mx; 2 Laboratorio Virtual NANOCOSMOS, Departamento de Medio Ambiente y Energía, Centro de investigación en Materiales Avanzados, Miguel de Cervantes 120, Complejo Industrial Chihuahua, Chihuahua, Chihuahua 31136, Mexico; daniel.glossman@cimav.edu.mx

**Keywords:** azomethine, DSSC, chemical hardness, free energy of electron injection

## Abstract

Ten molecules were theoretically calculated and studied through density functional theory with the M06 density functional and the 6-31G(d) basis set. The molecular systems have potential applications as sensitizers for dye-sensitized solar cells. Three molecules were taken from the literature, and seven are proposals inspired in the above, including the azomethine group in the π-bridge expecting a better charge transfer. These molecular structures are composed of triphenylamine (donor part); different combinations of azomethine, thiophene, and benzene derivatives (π-bridge); and cyanoacrylic acid (acceptor part). This study focused on the effect that the azomethine group caused on the π-bridge. Ground-state geometry optimization, the highest occupied molecular orbital, the lowest unoccupied molecular orbital, and their energy levels were obtained and analyzed. Absorption wavelengths, oscillator strengths, and electron transitions were obtained via time-dependent density functional theory using the M06-2X density functional and the 6-31G(d) basis set. The free energy of electron injection (ΔG_inj_) was calculated and analyzed. As an important part of this study, chemical reactivity parameters are discussed, such as chemical hardness, electrodonating power, electroaccepting power, and electrophilicity index. In conclusion, the inclusion of azomethine in the π-bridge improved the charge transfer and the electronic properties of triphenylamine-based dyes.

## 1. Introduction

**Figure 1:**
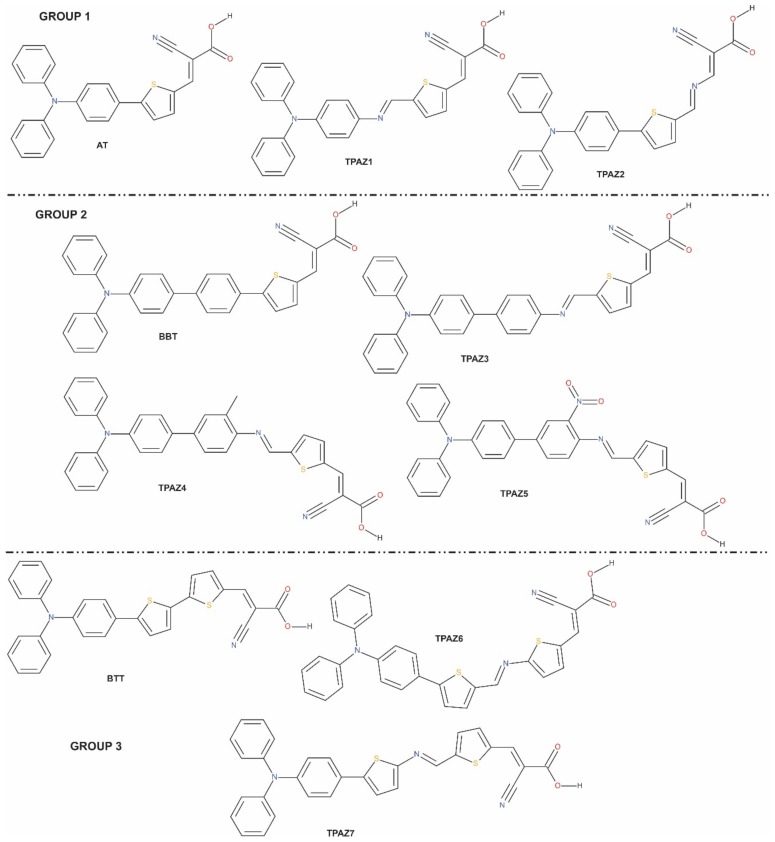
Molecular structures of triphenylamine-based dyes with different conjugation orders of the π-bride.

Solar energy is a form of renewable energy, being the most abundant on this planet and, thus, generating great interest to transform solar energy into electrical energy while being friendly to the environment. At present, different technologies are being developed to obtain higher efficiency. Silicon-based solar cells and dye-sensitized solar cells (DSSC) are some more developed devices for their promising efficiency; from the above, DSSCs have presented a great growth in efficiency in a short time. Grätzel in 1991 proposed the DSSC for the first time; since, it has been greatly studied [1]. DSSC is mainly composed of semi-conductive oxide, electrolyte, electrode, and sensitizer, which contribute to the cells’ performance. However, several authors have proposed that the cells’ efficiency can be modulated with the dye modification [2]. Recently, some dyes have been reported with high energy conversion efficiencies, such as ruthenium-based dyes up to 12% [3] and metal-free organic dyes up to 14.5% [4]. Metal-free organic dyes present some advantages because of their easy synthesis, low cost, and environmental friendliness [5]. On the other hand, dyes with donator π-bridge-acceptor (D-π-A) structure have reached high efficiencies [6]. Regarding D-π-A structure, several studies about dyes have been reported using different proposals. For example, modifying the donor part using coumarin [7,8], carbazole [9,10], phenothiazine [11,12], and triphenylamine [13,14]; modifying the acceptor part using cyanoacrylic acid [15] and alkoxysilane [16,17]; and mainly modifying the π-bridge using thiophene [18,19,20], benzene [21,22], dioxythiophene [23], and benzothiadiazole [24,25], among many others. Hence, the study and the understanding of the performance of D-π-A metal-free organic dyes is very important. The above can be reached via theoretical studies, which allows designing new dyes searching for the best efficiency. Specifically, Density Functional Theory (DFT) has been used to study the electronic properties of new and already reported dyes [26,27,28]. It is well known that the design of sensitizer dye consists mainly in modifying the π-bridge. On the other hand, several studies have reported the use of azomethine in molecular systems used in photovoltaics devices, finding an improvement in the luminescent properties and the molecular structures stability [29] and considering the azomethine as a photo-stable group [30] and with a good electric conductance [31]. Therefore, it is expected that azomethine is beneficial to charge transportation and has potential application to produce high-quality organic semiconductors [32]. Further, azomethine has been used in applications such as organic light-emitting diodes (OLEDs) [33,34,35], OFET [36,37], and DSSC [38,39]. Recently, Manzoor et al. reported a study of optical and photovoltaic properties of coumarin-based dyes with a similar azo group in the π-bridge, obtaining good absorption behavior in UV-visible region, good photovoltaic response, and a reduction in the HOMO-LUMO gap [7]. The main contribution of this paper is to study the effect of the azomethine group in sensitizers’ photoelectronic properties. To achieve the above, seven dyes with D-π-A structure were conformed by triphenylamine (TPA) in the donor part; cyanoacrylic acid in the acceptor part; and different conformations in the π-bridge using thiophene, benzene, methylbenzene, nitrobenzene, and azomethine groups. These dyes were inspired by three experimentally reported dyes, which are 2-Cyano-3-(5-(4-(diphenylamino)phenyl)thiophen-2-yl)acrylic acid [40], 2-Cyano-3-[5-[4′-(diphenylamino)[1,1′-biphenyl]-2-thienyl]-2-acrylic acid [41,42], and 2-Cyano-3-[5′-[4-(diphenylamino)phenyl][2,2′-bithiophen]-5-yl]-2-acrylic acid [41,43,44]. In this work, these dyes are called AT, BBT, and BTT, respectively. AT and BTT were theoretically studied by our work group [45]; however, these results are shown to compare to the seven studied dyes, which were named TPAZ identified from 1 to 7, as is shown in Figure 1. Through the Density Functional Theory (DFT), different optoelectronics properties were calculated of the already reported AT, BBT, and BTT dyes and of the TPA1, TPA2, TPA3, TPA4, TPA5, TPAZ6, and TPA7 dyes. The second group is inspired by the first group but includes the azomethine group in the π-bridge with different conjugations. The optimization of molecular geometry, the highest occupied molecular orbital (HOMO) and lowest unoccupied molecular orbital (LUMO) energy levels, the free energy of electron injection, UV-Vis absorptions and transitions, and chemical reactivity were evaluated and analyzed to compare both groups. Finally, the best sensitizers were chosen regarding their optoelectronic properties.

## 2. Results and Discussion

### 2.1. Molecular Structure of Dyes

The optimized structures reported correspond to the ground state in vacuum, which were obtained by M06/6-31G(d) level of calculation. In this work, seven molecules were studied with D-π-A structure. The π-bridge was conformed by two and three units of chemical groups such as thiophene, benzene, methylbenzene, nitrobenzene, and azomethine. Table 1 shows a summary of the most representative bond lengths and dihedral angles. Specifically, it reports the dihedral angles that are formed between i) the donor part and unit one of the π-bridge (D-π1); ii) unit one and unit two of the π-bridge (π1-π2); iii) unit two and unit three of the π-bridge (π2-π3); and iv) unit three of the π-bridge and the acceptor part (π3-A). Further, the bond lengths shown are those between the mentioned units. In Figure 1, it can be observed that the dyes reported in this research are grouped according to its similarities in chemical structure such that the main difference is the presence or the position of the azomethine group. For example, in group 1, TPAZ1 and TPAZ2 are similar to the AT dye; in group 2, TPAZ3, TPAZ4, and TPAZ5 are similar to BBT; and in group 3, TPAZ6 and TPAZ7 are similar to BTT. On the other hand, it can be observed that the presence of thiophene in π1 results in a structure more plane regarding D-π1 dihedral angle, as it occurs in AT, TPAZ2, BTT, TPAZ7, and TPAZ6. Further, in group 1, the presence of azomethine in π2 (TPAZ2) results in a structure more plane in π1-π2 than the AT dye. In group 2, the dihedral angle in D-π1 does not vary with the presence of the azomethine group, but in TPAZ3, the dihedral angle in π1-π2 varied 15° approximately. The rest of the dihedral angles did not present significant differences. In group 3, dihedral angles did not present significant differences between BTT, TPAZ6, and TPAZ7, but using azomethine decreases this angle. It is important to see that the difference between TPAZ6 and TPAZ7 is the position of the N atom in the azomethine, which resulted in a more planar geometry for TPAZ7, mainly in the π2-π3 angle. In general, it is expected that the dyes structural planarity is related to the improvements of charge transfer from donor moiety to the anchoring group [46]. Then, this property suggests that TPAZ2 and TPAZ7 are the best sensitizers.

### 2.2. Frontier Molecular Orbitals

**Figure 2:**
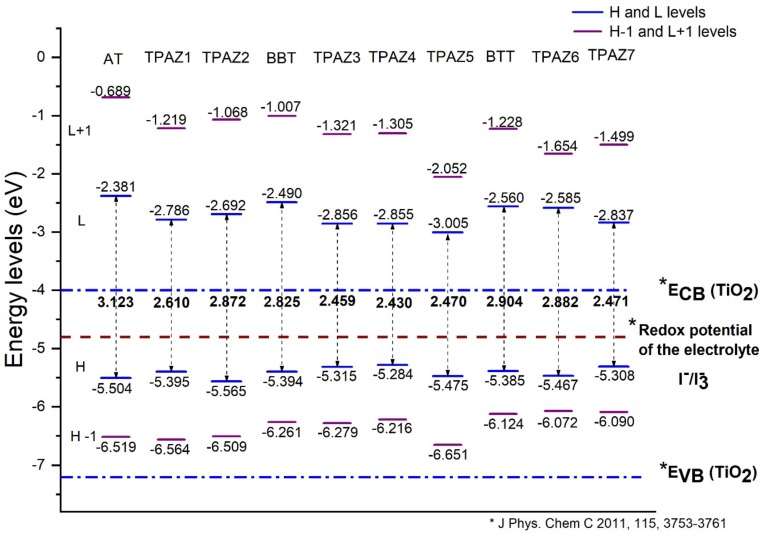
Highest occupied molecular orbital (HOMO) and lowest unoccupied molecular orbital (LUMO) energy levels of the triphenylamine-based dyes at M06/6-31G(d) level of theory.

The energy levels of the highest occupied molecular orbital (HOMO) and the lowest unoccupied molecular orbital (LUMO) were calculated with the M06/6-31G(d) level of calculation. The HOMO and LUMO energy levels are shown in Figure 2. For an efficient electron injection, the LUMO energy level must be above the band conduction of nanocrystalline semiconductor oxide (commonly used TiO_2_) [47,48], and for an efficient regeneration of the dye, the HOMO energy level must be below the redox potential of the electrolyte (commonly used $I^{-}/I_{3}^{-}$ redox couple) [49]. Furthermore, a smaller HOMO-LUMO gap is desired to sure an electron excitation from HOMO to LUMO with less energy. Figure 2 shows that the LUMO level of all dyes studied is above the conduction band of TiO_2_, which is located at −4.0 eV [50] and that the HOMO level of all dyes is below the redox potential of the electrolytes $I^{-}/I_{3}^{-}$ redox couple. Hence, the electron injection from the dye to the conduction band of TiO_2_ is thermodynamically favorable.

**Figure 3:**
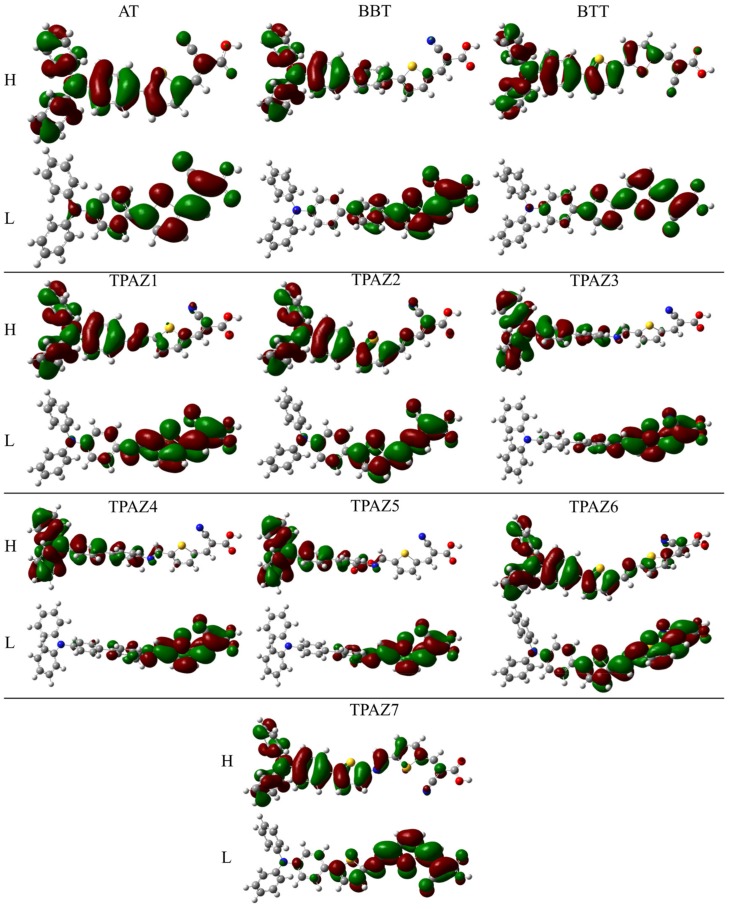
Density of HOMO and LUMO frontier molecular orbitals of the triphenylamine-based dyes at M06/6-31G(d) level of theory.

Also, it has been observed that the inclusion of the azomethine group induces more negative LUMO levels, namely, closer to the conduction band of TiO_2_ and, hence, with a more favorable electron injection. For example, in group 1, TPAZ1 (−2.786 eV) and TPAZ2 (−2.692 eV) have LUMO levels more negative than AT (−2.381 eV). Further, TPAZ1 has the LUMO level more negative than TPAZ2 and less gap (2.610 eV for TPAZ1 and 2.872 eV for TPAZ2). In group 2, TPAZ3 (−2.856 eV), TPAZ4 (−2.855 eV), and TPAZ5 (−3.005 eV) have LUMO levels more negative than BBT (−2.490 eV). Further, the dye with the methylbenzene group in π1 (TPAZ4) has the lowest gap ((2.430 eV) < TPAZ3 (2.459 eV) < TPAZ5 (2.470 eV) < BBT (2.825 eV)). In group 3, TPAZ6 (−2.585 eV) and TPAZ7 (−2.837 eV) have LUMO levels more negative than BTT (−2.560 eV). Further, TPAZ7 has the lowest gap ((2.471 eV) < TPAZ6 (2.882 eV) < BTT (2.904 eV)). In general, the LUMO levels closer to the conduction band of TiO_2_ are the sensitizer with benzene, methylbenzene, and nitrobenzene in π1, besides TPAZ7. These values are very similar: −3.005 eV to TPAZ5, −2.856 eV to TPAZ3, −2.855 eV to TPAZ4, and −2.837 eV to TPAZ7. Further, the HOMO-LUMO gap increases as follows: TPAZ4 (2.430 eV) < TPAZ3 (2.459 eV) < TPAZ5 (2.470 eV) < TPAZ7 (2.471 eV). The best dyes with regard to LUMO level and gap are TPAZ4, TPAZ3, TPAZ5, and TPAZ7. While that with regard to HOMO level are TPAZ4 (−5.284 eV), TPAZ7 (−5.308 eV) and TPAZ3 (−5.315 eV) because they are closer to redox potential of the electrolytes $I^{-}/I_{3}^{-}$ redox couple, which favors a better electron regeneration of the dye. Finally, it can be observed that the LUMO+1 and HOMO-1 levels have behaviors like that above. On the other hand, it is important to know that the mechanism of charge separation depends notably on the spatial distribution of the HOMO and LUMO density because it is significantly related to the electron injection. As shown in Figure 3, in all dyes, the HOMO density is mainly found on the triphenylamine part and the π-bridge and the LUMO density is mainly distributed on the π-bridge and cyanoacrylic acid part; the distribution meets the requirements according to the charge transfer mechanism. In addition, with the increase of π-conjugation of the linker by the inclusion of the azomethine group, a better load separation occurs as well as an adjustment in energy level values of HOMO and LUMO, causing a decrease in gap [51,52,53]. This occurred in groups 1, 2, and 3. Moreover, the best charge separation can be observed in TPAZ5, TPAZ4, and TPAZ3.

### 2.3. Ultraviolet-Visible Absorption Spectra

**Figure 4:**
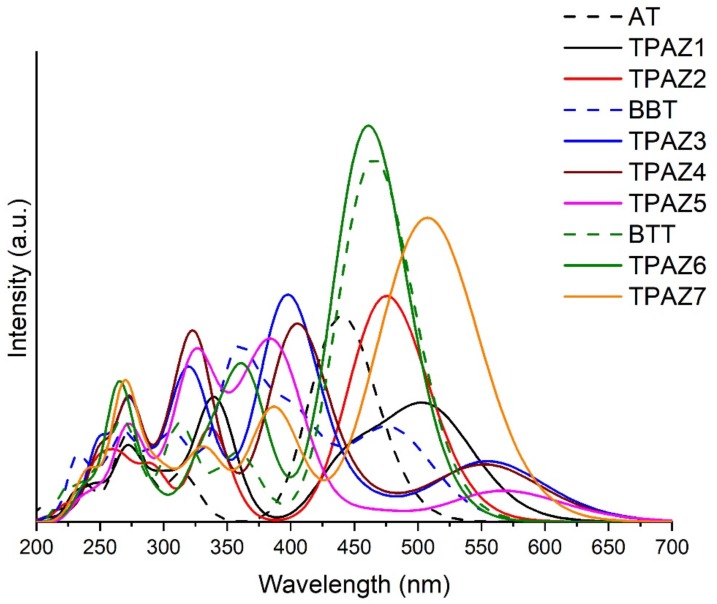
UV-Vis absorption spectra of triphenylamine-based dyes obtained with time-dependent Density Functional Theory (TD-DFT) and M06-2X/6-31G(d) level of theory.

Ultraviolet-Visible absorption spectra were calculated with the M06-2X/6-31G(d) level of calculation, using tetrahydrofuran (THF) as solvent. The calculation was carried out with nonequilibrium protocol, taking into account 20 excited states. Figure 4 shows the theoretical UV-Vis absorption spectra of the dyes. M06-2X/6-31G(d) level of calculation was validated by experimental UV-Vis spectra data, taking experimental values of maximum absorption wavelength (λ_max_) and comparing them with theoretical results, obtaining reasonable similarities (see Figure S1). Experimental λ_max_ taken from the bibliography were 415 nm [40], 417 nm [41], and 473 nm [44], while the theoretical λ_max_ obtained results of 446 nm, 486 nm, and 474 nm for AT, BBT, and BTT, respectively. All experimental and theoretical spectra were obtained in THF solvent. In Figure 4, it can be observed that the inclusion of the azomethine group in the π-bridge increases the conjugation of π bonds, which promotes a bathochromic displacement of λ_max_ [52,54,55,56,57,58], except in TPAZ6. Regarding the oscillator strength (f) reported in Table 2, a significant variation was not observed with the inclusion of azomethine. On the other hand, dyes with benzene-derived groups in π1 showed a bathochromic displacement of λ_max_ but also showed a hypochromic effect. For example, in group 2, TPAZ1 with azomethine in π1 presented λ_max_ of 513 nm and f value of 0.713 and TPAZ2 with thiophene in π1 showed λ_max_ of 483 nm and f value of 1.027. Meanwhile, TPAZ3 with benzene in π1 presented λ_max_ of 564 nm and f value of 0.340, TPAZ4 with methylbenzene in π1 presented λ_max_ of 563 nm and f value of 0.323, and TPAZ5 in π1 presented λ_max_ of 576 nm and f value of 0.206 (see Table 2). However, TPAZ3 and TPAZ4 showed higher f values from the secondary band of 0.878 and 0.719, respectively. All transitions of λ_max_ were from HOMO to LUMO (H-L). Group 2 had the higher H-L transitions of 92% in BBT, of 95% in TPAZ3, of 94% in TPAZ4, and of 96% in TPAZ5. According to λ_max_, the best dyes could be TPAZ5, TPAZ3, and TPAZ4. Additionally, other strong absorption bands were observed in the ultraviolet range with HOMO to LUMO+1 and HOMO-1 to LUMO transitions, among others (see Table 2).

The emission spectra were estimated through vertical excitation energy without considering external iteration calculations (these conditions were chosen taking into account the computational cost). Stokes shift can be consulted in the Supplementary Material (see Figure S1); the results indicate that the studied molecules constitute potential applications in organic light-emitting diodes (OLEDs), except TPAZ1 linking the azomethine group with the donor moiety.

### 2.4. Free Energy of Electron Injection

One of the important factors to predict the short-circuit current density (J_sc_) is the electron injection efficiency (Ø_inj_), which is influenced by the free energy of electron injection (ΔG_inj_), that is, Ø_inj_ α (−ΔG_inj_) [7,59,60]; therefore, Ø_inj_ is equal to the absolute values of ΔG_inj_. Thereby, the absolute values of ΔG_inj_ (Ø_inj_) for all dyes are much greater than 0.2 eV, as is shown in Table 3, and according to the literature [60], we can predict that these dyes have enough driving force for the fast injection of excited electrons from dyes to TiO_2_. The ΔG_inj_ oscillate between −1.23 and −0.68 eV, which is large enough to guarantee an efficient electron injection (AT, BBT, and BTT have been reported with good conversion efficiency [40,41,44]). Besides, if this ΔG_inj_ is too large, it may introduce energy redundancy and result in a smaller open-circuit voltage (V_oc_) and large thermalization losses [61,62]. Furthermore, ΔG_inj_ increases in the order: AT (−1.28) < BTT (−1.23) < TPAZ6 (−1.17) < BBT (−1.16) < TPAZ7 (−1.07) < TPAZ1 (−1.02) < TPAZ2 (−1.01) < TPAZ4 (−0.92) < TPAZ3 (−0.89) < TPAZ5 (−0.68). The results reflect that all the dyes have good electron injection efficiencies with AT, BTT, TPAZ6, BBT, and TPAZ7 having better electron injection efficiencies. Despite TPAZ4, TPAZ3, and TPAZ5 presenting the lowest electron injection efficiencies, it should be considered that this study considers as a parameter the oscillator strength of λ_max_; however, these dyes have other absorption bands with a high oscillator strength.

On the other hand, the f parameter is related to light harvesting efficiency (LHE) [63,64,65], and higher values indicating that short-circuit photocurrent (J_sc_) can be increased [66]. However, according to LHE, the best dyes could be TPAZ6 with 0.94, BTT with 0.93, TPAZ7 with 0.91, TPAZ2 with 0.906, and AT with 0.88. Note that the best electron conjugation occurs when the thiophene group is next to the donators zone; see Table 3. It should be noted that this parameter only contemplates a wavelength (λ_max_) and does not represent all others that also contribute to the absorption of the sensitizer, and so, they should be considered in light harvesting.

### 2.5. Chemical Reactivity Parameters

**Figure 5:**
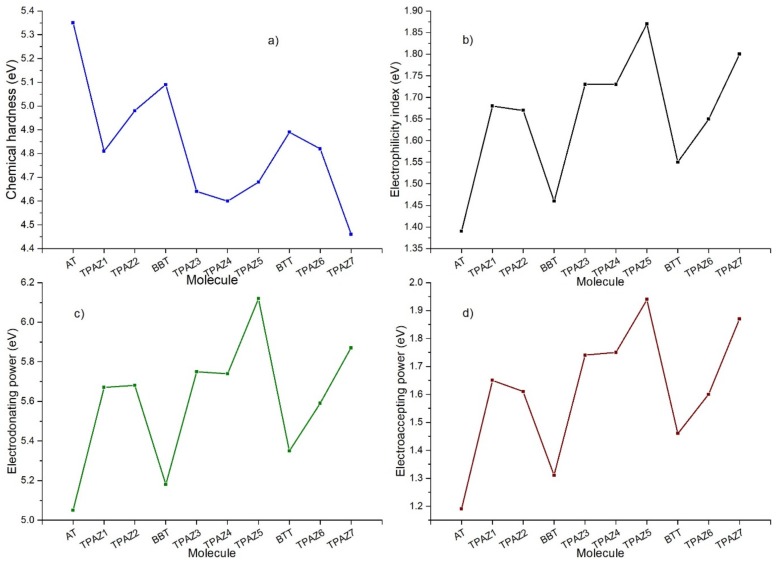
(**a**) Chemical hardness, (**b**) electrophilicity index, (**c**) electrodonating power, and (**d**) electroaccepting power of the triphenylamine based dyes at M06/6-31G(d).

Chemical reactivity parameters were calculated through DFT conceptual using M06/6-31G(d) level of calculation to obtain neutral and ionic energies. Chemical hardness (η), electrophilicity index (ω), electrodonating power (ω^−^), and electroaccepting power (ω^+^) were obtained and analyzed. The chemical hardness has been related to the ease with which electrons are transferred through the molecule [67,68]. This parameter, furthermore, has been inversely associated with the conversion efficiency of the DSSC, which is highly supported on correlation calculations between theoretical results of the chemical hardness and experimental data of the efficiency [69]. Likewise, the electrodonating power and electroaccepting power are related to the capacity of the molecule to donate and accept electrons, respectively [70], and the electrophilicity index is related to the stabilizing energy that a system experiences when it is saturated with electrons [71]. Mainly, lower values of chemical hardness are expected to consider the highest efficiency [72]. Furthermore, higher values of electrophilicity and electroaccepting power are expected to consider the highest efficiency in dyes with the modification in the π-bridge (see Figure 5).

Table 4 shows the calculated chemical reactivity parameters. It can be observed that the inclusion of the azomethine group in the π-bridge decreases in the chemical hardness in each group of dyes. Further, it increases in electrophilicity index, electrodonating power, and electroaccepting power. The lower values of chemical hardness resulted in the next order: TPAZ7 (4.46 eV) < TPAZ4 (4.60 eV) < TPAZ3 (4.63 eV) < TPAZ5 (4.68 eV), which have the azomethine group between the π-bridge units (π1-π3). Other dyes had the next values of chemical hardness: TPAZ1 (4.81 eV) < TPAZ6 (4.82 eV) < TPAZ2 (4.98 eV). The highest values in electrophilicity index, electrodonating power, and electroaccepting power were present in decreasing order as TPAZ5 > TPAZ7 > TPAZ4 > TPAZ3 > TPAZ1 > TPAZ2 > BTT > BBT > AT. The lowest chemical hardness and the highest electrophilicity index, electrodonating power, and electroaccepting power were obtained by the dyes with the inclusion of azomethine.

According to the analyzed properties, the best molecules to be used as sensitizer are TPAZ7, TPAZ4, TPAZ5, and TPAZ3, with similar optoelectronic properties among them. Further, this group of dyes presented optoelectronic properties far better than AT, BBT, and BTT, which have been reported with high efficiency.

## 3. Computational Details

A theoretical study was carried out on the optoelectronic properties of the proposed molecular systems. Ground state molecular structure was obtained using Density Functional Theory (DFT) [73,74] with the M06 hybrid meta-GGA density functional [75] combined with the 6-31G(d) [76,77] basis set, proposed by Pople. Frequencies were reviewed to guarantee the non-presence of the imaginary frequencies, namely, to guarantee the molecular structure of the global minimum energy. Likewise, energy levels and electron density of the highest occupied molecular orbital (HOMO) and the lowest unoccupied molecular orbital (LUMO) and chemical reactivity parameters were obtained. Chemical reactivity parameters were obtained with DFT conceptual by ionic and neutral energy calculations, such as chemical hardness (η) [78], electrodonating power (ω^−^) and electroaccepting (ω^+^) power [70], and electrophilicity index (ω) [71]. Ultraviolet-visible (UV-vis) absorption spectra were calculated using time-dependent DFT (TD-DFT) [79,80] with M06-2X hybrid meta-GGA density functional [75] combined with 6-31G(d) [76,77] basis set to obtain maximum absorption wavelength (λ_max_). UV-Vis spectra were calculated by nonequilibrium protocol [81,82]; tetrahydrofuran (THF) was considered as solvent; and its effect was calculated through integral equational formalism polarizable continuum model (IEF-PCM) [83], an implicit method. The equations were resolved for 20 excited states. Absorption spectra data was processed using the Swizard program [84] and the Gaussian model to read oscillator strength (f) and orbitals involved in the electron transition. Also, the free energy of electron injection (ΔG_inj_) was obtained for all molecules, this being the difference between oxidation potential energy of the excited state (Eox^dye*^) and the reduction potential energy of TiO_2_ conduction band (ECB = −4.0 eV). Then, it is expressed as ΔG_inj_ = Eox^dye*^ − ECB and, successively, as Eox^dye*^ = Eox^dye^ − ΔE, where Eox^dye^ is the ground-state oxidation potential (−HOMO) of the dye and ΔE is the absorption energy in eV associated with λ_max_ (vertical excitation energy of λ_max_); for more details, consult References [7,59,60]. The light harvesting efficiency (LHE) was obtained by $LHE\left(\lambda \right)=1-10^{-f}$, where f is the oscillator strength associated to λ_max_ [26,85,86]. All calculations were carried out using the Gaussian 09 Revision D.01 [87].

## 4. Conclusions

We have presented the study of 10 dyes, of which three have already been reported experimentally and theoretically and of which seven are new structures inspired by the former. The effect of the π-bridge was evaluated by combining azomethine, thiophene, and benzene derivatives using two and three units. In all cases, the inclusion of azomethine improved the electronic properties such as UV-Vis absorption, charge transfer from the donator part to acceptor part and the electron injection according to HOMO and LUMO levels, and chemical reactivity. Therefore, considering the obtained results, the dyes with the best properties are TPAZ7, TPAZ4, TPAZ3, and TPAZ5. The chemical hardness received particular attention in this prediction regarding previous studies reported [5,88,89,90]. It can be recommended to synthesize and experimentally research dyes with azomethine on the π-bridge in DSSC.

## Figures and Tables

**Table 1:** Resume of bond lengths (Å) and dihedral angles (degrees) of the calculated dyes at M06/6-31G(d) level of theory.

Dyes	Donor Part (D)	π-Bridging		Acceptor Part (A)
	D-π1	π1-π2	π2-π3	π3-A
AT				
Dihedral	22.5	−	−	−0.9
Distance	1.45	−	−	1.42
TPAZ1				
Dihedral	−26.8	−1.4	−	−0.2
Distance	1.39	1.45	−	1.42
TPAZ2				
Dihedral	−19.0	0.5	−	0.1
Distance	1.45	1.42	−	1.36
BBT				
Dihedral	−33.1	23.2	−	−0.8
Distance	1.47	1.46	−	1.42
TPAZ3				
Dihedral	−33.8	−32.1	−1.2	−0.1
Distance	1.47	1.40	1.45	1.42
TPAZ4				
Dihedral	−33.6	−34.9	−1.0	0.1
Distance	1.47	1.40	1.45	1.42
TPAZ5				
Dihedral	−33.3	−49.1	4.9	0.4
Distance	1.47	1.39	1.45	1.42
BTT				
Dihedral	−23.5	7.7	−	−0.3
Distance	1.45	1.45	−	1.42
TPAZ6				
Dihedral	−22.7	−0.7	−30.3	−0.3
Distance	1.46	1.43	1.37	1.42
TPAZ7				
Dihedral	−19.5	1.9	0.6	0.1
Distance	1.45	1.36	1.43	1.42

**Table 2:** Absorption wavelengths, vertical excitation energy (*E*), oscillator strengths (f), and the orbitals involved in the transitions of triphenylamine-based dyes at M06-2X/6-31G(d) level of theory.

Molecule	λ_max_ (nm)	*E* (eV)	f	Transitions H = HOMO, L = LUMO (%)
AT	446	2.78	0.928	H → L (90%)
	315	3.94	0.260	H-1 → L (84%)
	282	4.40	0.169	H → L+1 (72%)
	265	4.67	0.198	H → L+3 (89%)
TPAZ1	513	2.42	0.713	H → L (90%)
	342	3.63	0.624	H-1 → L (66%)
	306	4.05	0.192	H → L+1 (66%)
	268	4.63	0.234	H → L+4 (55%) H → L+5 (35%)
TPAZ2	483	2.57	1.027	H → L (89%)
	338	3.67	0.492	H-1 → L (82%)
	297	4.17	0.138	H → L+1 (54%) H-9 → L (25%)
	263	4.71	0.140	H → L+4 (67%)
BBT	486	2.55	0.520	H → L (92%)
	361	3.43	0.869	H-1 → L (71%)
	315	3.94	0.333	H → L+1 (71%)
	233	5.32	0.208	H-1 → L+1 (72%)
TPAZ3	564	2.20	0.340	H →L (95%)
	395	3.14	0.878	H-1 → L (65%)
	331	3.75	0.228	H → L+1 (69%)
	322	3.85	0.423	H-8 → L (42%) H-6 → L (23%)
	269	4.61	0.237	H → L+7 (88%)
TPAZ4	563	2.20	0.323	H → L (94%)
	403	3.08	0.719	H-1 → L (67%)
	329	3.77	0.288	H → L+1 (68%)
	325	3.81	0.518	H-8 → L (42%) H-7 → L (27%)
	269	4.61	0.205	H → L+7 (72%)
TPAZ5	576	2.15	0.206	H → L (96%)
	388	3.20	0.536	H → L+1 (56%)
	372	3.33	0.313	H → L+1 (32%) H-1 → L (21%)
	331	3.75	0.392	H → L+2 (49%) H → L+3 (27%)
	326	3.80	0.411	H-6 → L (42%) H-7 → L (32%)
	267	4.64	0.233	H → L+9 (92%)
BTT	474	2.62	1.148	H → L (80%)
	360	3.44	0.303	H-1 → L (77%)
	316	3.92	0.311	H → L+1 (77%)
	269	4.61	0.212	H → L+4 (88%)
TPAZ6	469	2.64	1.225	H → L (74%)
	366	3.39	0.545	H-1 → L (68%)
	338	3.67	0.261	H → L+1 (74%)
	268	4.63	0.213	H → L+4 (83%)
TPAZ7	522	2.38	1.036	H → L (84%)
	390	3.18	0.501	H-1 → L (76%)
	337	3.68	0.230	H → L+1 (74%)
	269	4.61	0.185	H → L+5 (72%)

**Table 3:** Ground-state oxidation potential energy (Eox^dye^), absorption energy associated with λ_max_ (ΔE), oxidation potential energy of the excited state (Eox^dye*^), driving force of electron injection (ΔG_inj_), and light harvesting efficiency (LHE).

Molecule	Eox^dye^ (eV)	∆E(eV)	Eox^dye*^ (eV)	∆G_inj_ (eV)	LHE
AT	5.50	2.78	2.72	−1.28	0.88
TPAZ1	5.40	2.42	2.98	−1.02	0.81
TPAZ2	5.56	2.57	2.99	−1.01	0.91
BBT	5.39	2.55	2.84	−1.16	0.70
TPAZ3	5.31	2.20	3.11	−0.89	0.54
TPAZ4	5.28	2.20	3.08	−0.92	0.53
TPAZ5	5.47	2.15	3.32	−0.68	0.38
BTT	5.39	2.62	2.77	−1.23	0.93
TPAZ6	5.47	2.64	2.83	−1.17	0.94
TPAZ7	5.31	2.38	2.93	−1.07	0.908

**Table 4:** Chemical reactivity parameters of triphenylamine based dyes (in eV) obtained by DFT conceptual at M06/6-31G(d) level of theory.

Molecule	η	ω	ω^−^	ω^+^
AT	5.35	1.39	5.05	1.19
TPAZ1	4.81	1.68	5.67	1.65
TPAZ2	4.98	1.67	5.68	1.61
BBT	5.09	1.46	5.18	1.31
TPAZ3	4.65	1.73	5.75	1.74
TPAZ4	4.60	1.73	5.74	1.75
TPAZ5	4.68	1.87	6.12	1.94
BTT	4.89	1.55	5.35	1.46
TPAZ6	4.82	1.65	5.59	1.60
TPAZ7	4.46	1.80	5.87	1.87

## Supplementary Materials

The following are available online, Figure S1: Comparison of UV–Vis absorption spectra of triphenylamine-based dyes. Experimental λmax was taken from the bibliography and theoretical results obtained with TD-DFT and M06-2X/6-31G(d) level of theory, Table S1: Stokes shift of triphenylamine based dyes at M06-2X/6-31G(d) level of theory.
