# Supplementary material for: Theoretical Study of the Effect of Different π Bridges Including an Azomethine Group in Triphenylamine-Based Dye for Dye-Sensitized Solar Cells
Source: Molecules. 2019 Oct 29;24(21):3897. doi: 10.3390/molecules24213897 (PMC6864646; doi:10.3390/molecules24213897)
Supplement: Supplementary file 1 [file molecules-24-03897-s001.pdf]

Supplementary Material

Theoretical study of the effect of different π bridges including an azomethine group in triphenylamine based dye for dye-sensitized solar cells

Tomás Delgado-Montiel [1], Rody Soto-Rojo [1,*], Jesús Baldenebro-López [1,*] and Daniel Glossman-Mitnik [2]

[1] Facultad de Ingeniería Mochis, Universidad Autónoma de Sinaloa. Prol. Ángel Flores y Fuente de Poseidón, S/N, 81223, Los Mochis, Sinaloa; México.

[2] Laboratorio Virtual NANOCOSMOS, Departamento de Medio Ambiente y Energía, Centro de investigación en Materiales Avanzados, Miguel de Cervantes 120, Complejo industrial Chihuahua, Chihuahua, Chihuahua, 31136, México.

* Correspondence: rody.soto@uas.edu.mx (R.S.-R); jesus.baldenebro@uas.edu.mx (J.B.-L)

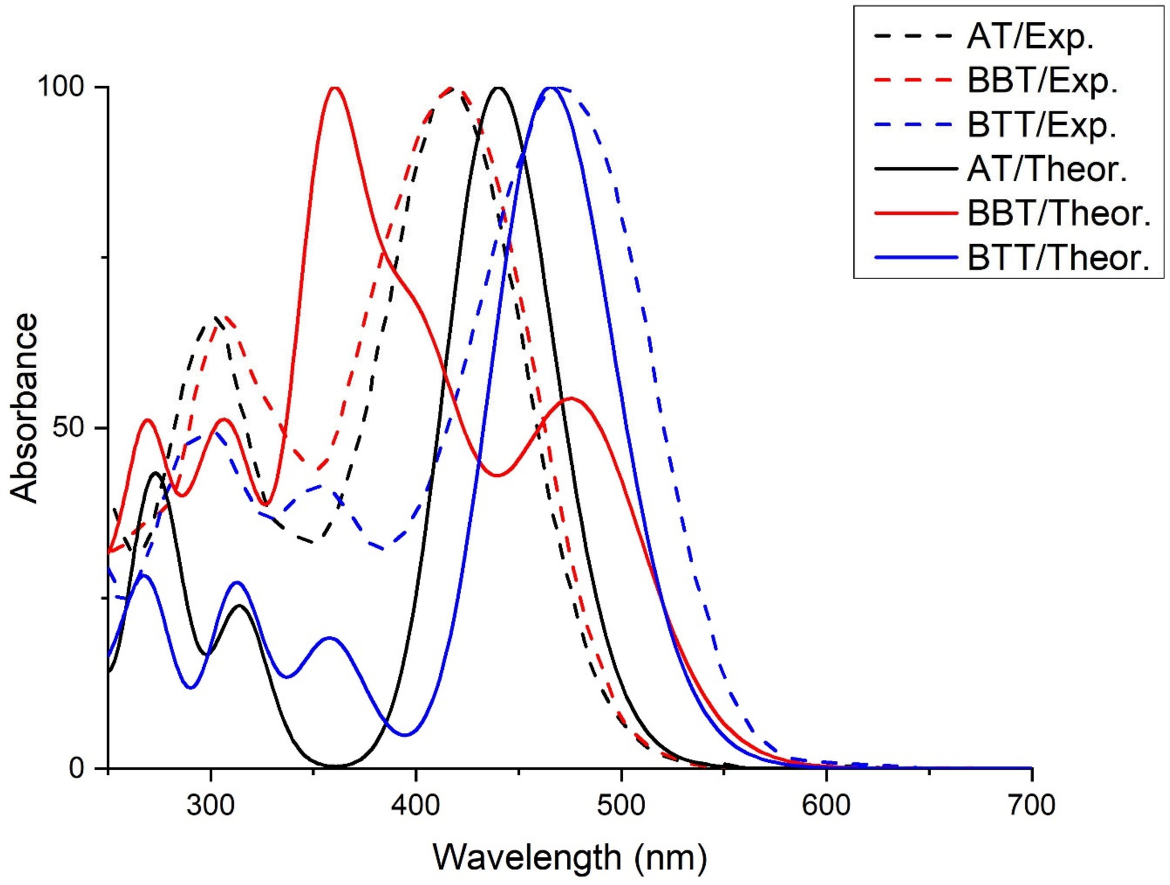

Figure S1. Comparison of UV–Vis absorption spectra of triphenylamine-based dyes. Experimental λmax was taken from the bibliography and theoretical results obtained with TD-DFT and M06-2X/6-31G(d) level of theory.

The experimental values were added with the permission of the authors, adding the citation of the bibliography. For a better appreciation of the results, these were normalized.

Table S1. Stokes shift of triphenylamine based dyes at M06-2X/6-31G(d) level of theory.

Molecule	Stokes Shift (nm)
AT	66
TPAZ1	-
TPAZ2	70
BBT	82
TPAZ3	159
TPAZ4	164
TPAZ5	194
BTT	72
TPAZ6	102
TPAZ7	104

* TPAZ1 does not present emission.
